# Natural Occurrence of Mycotoxin-Producing *Fusaria* in Market-Bought Peruvian Cereals: A Food Safety Threat for Andean Populations

**DOI:** 10.3390/toxins13020172

**Published:** 2021-02-23

**Authors:** Christine Ducos, Laetitia Pinson-Gadais, Sylvain Chereau, Florence Richard-Forget, Pedro Vásquez-Ocmín, Juan Pablo Cerapio, Sandro Casavilca-Zambrano, Eloy Ruiz, Pascal Pineau, Stéphane Bertani, Nadia Ponts

**Affiliations:** 1 INRAE, MycSA, F-33882 Villenave d’Ornon, France; christine.ducos@inrae.fr (C.D.); laetitia.pinson-gadais@inrae.fr (L.P.-G.); sylvain.chereau@inrae.fr (S.C.); florence.forget@inrae.fr (F.R.-F.); 2 Université de Toulouse, IRD, UPS, UMR 152 PHARMADEV, 31000 Toulouse, France; pedro.vasquez-ocmin@ird.fr; 3 Unité Organisation Nucléaire et Oncogenèse, Institut Pasteur, UPMC Univ. Paris 06, Sorbonne Universités, 75015 Paris, France; juan-pablo.cerapio-arroyo@inserm.fr; 4 Instituto Nacional de Enfermedades Neoplásicas, Departamento de Patología, Lima 15038, Peru; scasavilca@inen.sld.pe; 5 Instituto Nacional de Enfermedades Neoplásicas, Departamento de Cirugía en Abdomen, Lima 15038, Peru; ruizeloy@gmail.com; 6 Institut Pasteur, Unité Organisation Nucléaire et Oncogenèse, INSERM, U 993, 75015 Paris, France; pascal.pineau@pasteur.fr

**Keywords:** Fumonisins B, Peru, corn

## Abstract

Consumption of cereals contaminated by mycotoxins poses health risks. For instance, Fumonisins B, mainly produced by *Fusarium verticillioides* and *Fusarium* *proliferatum*, and the type B trichothecene deoxynivalenol, typically produced by *Fusarium graminearum,* are highly prevalent on cereal grains that are staples of many cultural diets and known to represent a toxic risk hazard. In Peru, corn and other cereals are frequently consumed on a daily basis under various forms, the majority of food grains being sold through traditional markets for direct consumption. Here, we surveyed mycotoxin contents of market-bought grain samples in order to assess the threat these mycotoxins might represent to Peruvian population, with a focus on corn. We found that nearly one sample of Peruvian corn out of six was contaminated with very high levels of Fumonisins, levels mostly ascribed to the presence of *F. verticillioides*. Extensive profiling of Peruvian corn kernels for fungal contaminants could provide elements to refine the potential risk associated with *Fusarium* toxins and help define adapted food safety standards.

## 1. Introduction

Cereals are frequently contaminated by mycotoxins [1], which are secondary metabolites produced by fungi with adverse effects on human and animal health upon ingestion. A recent survey reported that more than two-third of diverse cereal grains sampled in various regions of the world were co-contaminated by two or more families of mycotoxins [2]. Fumonisins B (or FB, mainly produced by *Fusarium verticillioides* and *Fusarium proliferatum*, see Reference [3] for a review) and the type B trichothecene (TCTB) deoxynivalenol (or DON, typically produced by *Fusarium graminearum* [4,5]) were found abundantly prevalent on cereal grains of all continents but Oceania, and under all latitudes including South America [2]. *F. graminearum* is also a producer of the estrogen-like mycotoxin Zearalenone (ZEN), frequently found worldwide (reviewed in Reference [6]). Considering Fumonisins, the form B1 (FB1) is the major one produced, usually co-occurring with smaller quantities of the forms B2 (FB2) and B3 (FB3). The *Fusarium* toxins Fumonisins, DON, ZEN, and T-2 toxin (Trichothecene A family) were recently reported as the most prevalent mycotoxins found in cereals in South America (69%, 53%, 43%, and 31%, respectively [2]). Toxicities of major toxins produced by *Fusarium* spp. are demonstrated, including carcinogenic activity for a number of them such as Fumonisins and DON (see References [7,8,9,10,11] for recent reviews). On corn specifically, 2019/2020 surveys in South America (including Peru) highlight that maize kernels are predominantly contaminated with *Fusarium* mycotoxins, Fumonisins always being the major contaminant [12,13].

**Figure 1:**
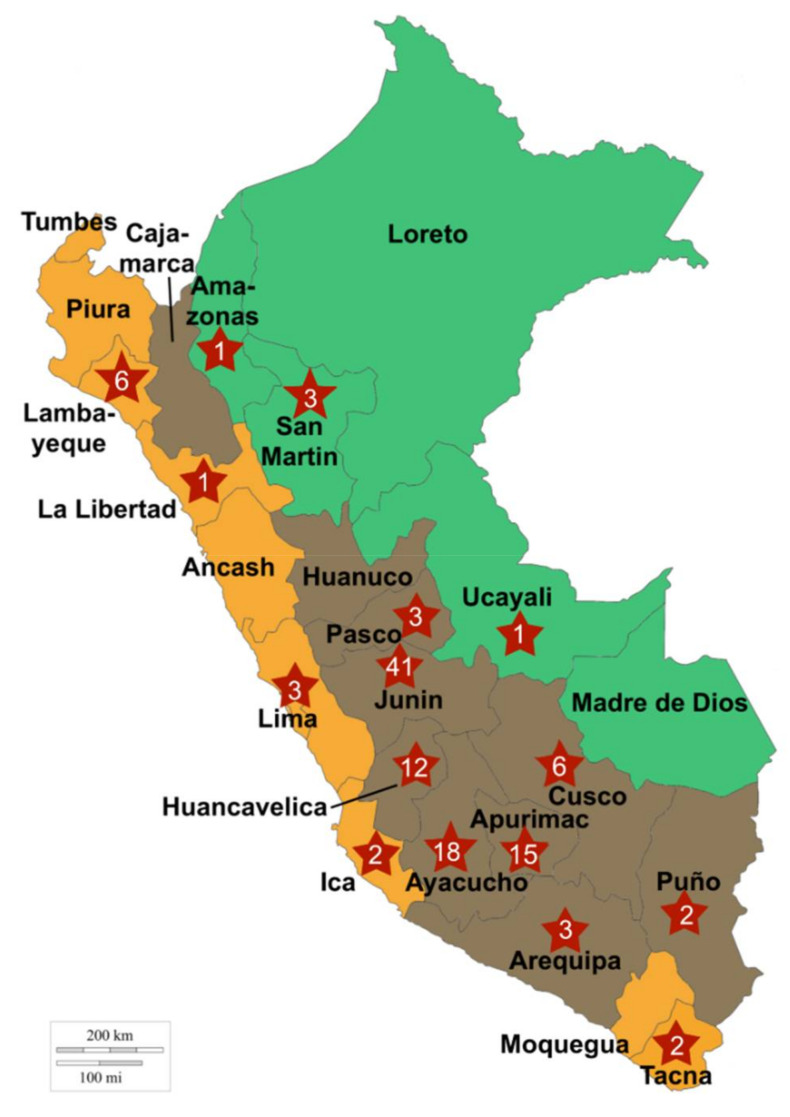
Geographical origins of the collected samples. The names of the 24 administrative departments of Peru are indicated in black. Department of collections is stamped with a red star marked with the number of samples collected. Green = *Selva*; brown = *Sierra*; orange = *Costa*.

Peruvian food production includes, in particular, corn (including purple and yellow corn used in the preparation of beverages widely consumed locally, i.e., *chicha morada* and *chica de jora*, respectively), rice, and other cereals. Wheat (essentially bread wheat) is a minor crop mostly cultivated in the southern Andean areas. Produced wheat and wheat flour are mostly consumed locally either directly (soup, purees) or for baking. Barley is also often consumed, although at much lower levels than corn, rice, and wheat (more than 10 times less than corn). In addition, the traditional Andean cereal-like grains quinoa (*Chenopodium quinoa*), kiwicha (*Amaranthus caudatus*), and kañiwa (*Chenopodium pallidicaule*) are frequently consumed, although most of the quinoa production is intended to exportation. In Peru, the majority of food grains are sold through traditional markets for direct consumption. Farming systems are extremely diverse and vary according to the anthropogenic biome considered, i.e., *Costa* (highly populated coastal regions), *Sierra* (Andean highlands with rural communities), and *Selva* (sparsely populated Amazon rainforest zones) (Figure 1) [14]. In the *Costa* region and its favorable climate condition, farming is organized in highly productive well-organized intensive systems located in the vicinity of major consumption centers and export points [15]. In the *Sierra* region, farming systems are mostly small-scale, with crop fields usually dispersed and fragmented into small surfaces. Production is subsistence-oriented, with about 60% of the production meant for self-consumption, and crops that are staples of the Peruvian diet (corn, wheat, and pseudo-cereal Andean grains such as quinoa) are produced at intermediate altitudes. Yields are usually low in the Sierra and the Selva regions. Recently, concerns were raised regarding weak food safety and quality standards in the Peruvian fruit and vegetable food chain, limiting access to international markets and impacting negatively public health [16]. Considering the above reported large prevalence of *Fusarium* toxins DON and FB in cereals, including in South America, a survey was conducted in order to assess the threat these mycotoxins might represent in Peru, specifically, with a focus on corn.

## 2. Results

### 2.1. Occurrence of Fusarium spp. in Peruvian Cereals Destined for Direct Consumption

A total of 119 cereal and pseudo-cereal samples—consisting of corn, wheat, amaranth grains (quinoa, kañiwa, and kiwicha), rice, barley, and oat—were collected from local Peruvian markets located in different areas of the country between 2014 and 2016 (Table 1).

As mentioned above, Peru can be divided into three main landscapes: the Costa (coastal landscapes running along the pacific coastline), the Selva (eastern jungle-type landscapes along the borders with Ecuador, Colombia, and Brazil), and the Sierra that follows the Andes and contains most of the cultivated lands (Figure 1). Most samples (84%) originated from various Andean regions of Peru (Sierra), where cereals and pseudo-cereals are mainly produced. The majority of samples consisted of corn kernels (50.4%), followed by wheat (21%) and amaranth seeds and rice (10.1% each).

**Figure 2:**
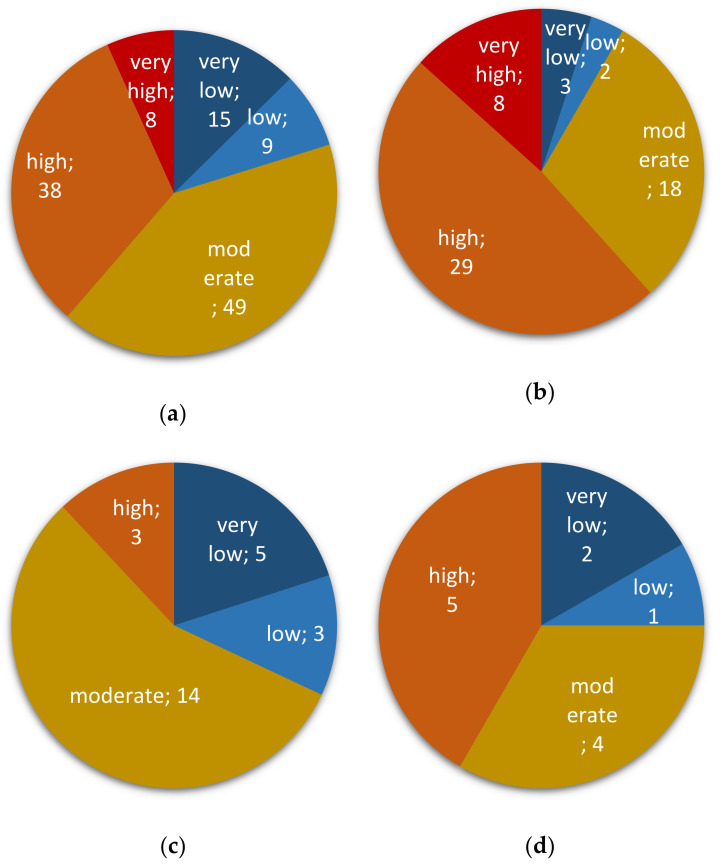
*Fusarium* risk assessment in collected cereal and pseudo-cereal grains. Risk is ranked as *very low*, *low*, *moderate*, *high*, or *very high*. Number after semicolon indicates corresponding sample size. (**a**) all sample types; (**b**) corn; (**c**) wheat; (**d**) rice.

All samples were tested for the presence of fungal species belonging to the genus *Fusarium* by qPCR detection of a conserved region of the ß-tubulin chain gene (see the Materials and Methods section). Using an estimate of the abundance of the target by qPCR, mycotoxin risk levels related to the presence and relative abundance of *Fusarium* spp. were further graded “very low” (when *Fusarium* presence was not detected; see Materials and Methods), “low”, “moderate”, “high”, or “very high”. A total of 104 grain samples (85% of them) were tested positive for the presence of *Fusarium* spp. (low to very high risk; Figure 2a): 57 of corn (95% of corn samples), 20 of wheat (80%), 11 of amaranth grains (92%), 10 of rice (83%), two of barley (33%), and four of oat out of four tested (Figure 2 and Figure S1). Ninety-five samples (80%) were found at moderate to very high risk to be contaminated with mycotoxins produced by *Fusarium* spp. (Figure 2a). All samples found at very high-risk levels and most samples rated as high risk (29 out of 38) were of corn (Figure 2b). With very frequent contaminations at moderate to very high levels, corn kernels presented the highest mycotoxin risk associated with the presence of *Fusarium* species (Figure 2b). Wheat, rice, and amaranth grains were next in the ranking of contamination frequencies, mostly at moderate levels for wheat and amaranth grains (Figure 2c and Figure S1a, respectively) and high and moderate for rice (Figure 2d). Barley and oat samples were mostly qualified with low to very low *Fusarium*-associated risks (Figure S1b,c).

### 2.2. Assessing Contaminations with Fumonisins and Fumonisin-Producing Fusarium Species in Crop Samples

The risk of having *Fusarium* toxin contaminants in food samples can be assessed by the quantitative detection of one or more genes mandatory for their biosynthesis in DNA extracted from the kernels to be investigated. If the target gene is not detected, then the sample is likely free of the corresponding toxin. Conversely, a positive assay indicates one or more fungi that have the capacity to produce this toxin are present, and thus a risk of kernel contamination with toxins. Similar to the method used to estimate risks associated with the presence of Fusaria in samples, qPCR assays on such target genes were used to qualify the corresponding mycotoxin-associated risks as *low*, *moderate*, *high,* or *very high*.

**Figure 3:**
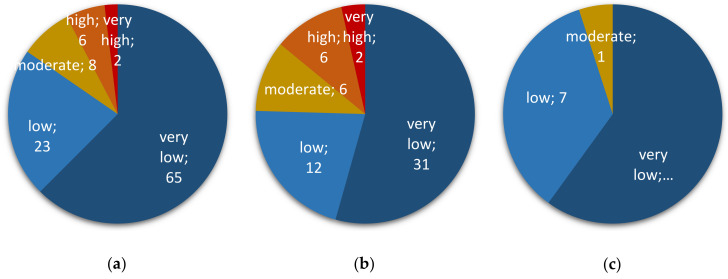
*Fumonisin* risk assessment in collected cereal and pseudo-cereal grains. Risk is ranked as *very low*, *low*, *moderate*, *high*, or *very high*. Number after semicolon indicates corresponding sample size. (**a**) all sample types; (**b**) corn; (**c**) wheat.

To assess fumonisin-related risks, we targeted the gene *Fum1*, a major gene involved in the production of Fumonisins B (including FB1, FB2, and FB3) [17]. Out of the 104 samples positive for the presence of *Fusarium* spp., 39 (38% of them) were positive for the presence of *Fum1* (Figure 3a): a little under half of the corn samples (46%; 26 out of 57; Figure 3b), 40% of the wheat samples (eight out of 20; Figure 3c), less than a third of the rice samples (30%; three out of 10), and one sample of oat or amaranth seeds (out of four or 11, respectively) (Figure S2). None of the two barley samples were tested positive for *Fum1*. All crops considered, fumonisin-associated risk largely came from corn samples with 26 corn samples out of the 39 positive ones for *Fum1* (i.e., 25% of the samples that tested positive for *Fusarium* spp.).

**Figure 4:**
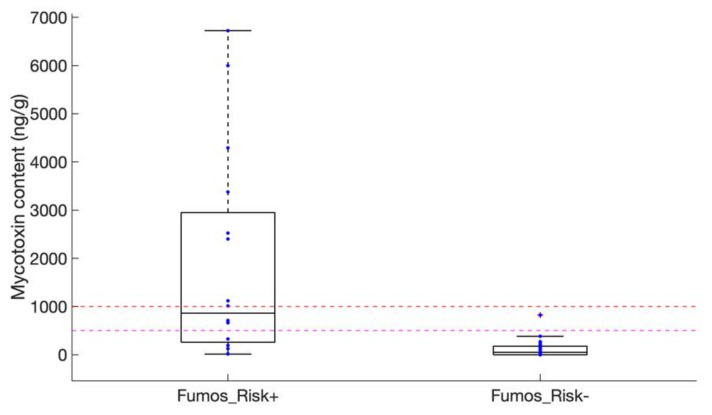
Boxplot representation of *Fumonisin* contents of selected samples. “Fumos_Risk+” refers to samples with fumonisin-associated risk ranked as *moderate* to *very high*; “Fumos_Risk-” refers to samples with risk ranked as *very low* to *low*. The pink and red dashed lines indicate the 500 and 1000 ng·g^−1^ thresholds, respectively.

Samples with risk categorized *moderate* to *very high* (14 samples of corn, one of rice, and one of wheat) were further analyzed for the presence of Fumonisins B1, B2, and B3 by analytical methods (HPLC-FLD, see Materials and Methods). All showed some level of contamination, ranging from trace amounts of total Fumonisins to 6725 ng of total Fumonisins per g of dry matter (ng·g^−1^; Figure 4). FB1 was always found as the most abundant fumonisin (data not shown). Total fumonisin content exceeded 1000 ng·g^−1^, i.e., the maximum acceptable level of contamination for corn destined to direct human consumption as defined by European Regulations (EC 1881/2006), in 8 of these 16 samples categorized at *moderate* to *very high* risk, all of them being corn samples (three additional ones being contaminated at levels above 500 ng·g^−1^). A positive correlation (estimated R^2^ = 0.604 and *p*-value = 0.013) could further be detected between fumonisin content and the relative abundance of *Fum1*-containing DNA measured by qPCR (Figure S3). We supported this observation by measuring fumonisin contents in 36 randomly selected samples out of the 88 ones with risk categorized low to very low. A total of 14 and six samples contained no detectable levels or trace amounts, respectively, of Fumonisins, and only one sample of corn was contaminated with 826 ng·g^−1^ of total Fumonisins (Figure 4). Altogether, our results underline the pertinence of using rapid DNA-based qPCR methods for evaluating *Fusarium* contaminations in cereals. Our analysis further revealed that nearly one sample of Peruvian corn out of six was contaminated with very high levels of Fumonisins, well exceeding the 1000 ng·g^−1^ maximum admissible level for safe human consumption.

**Figure 5:**
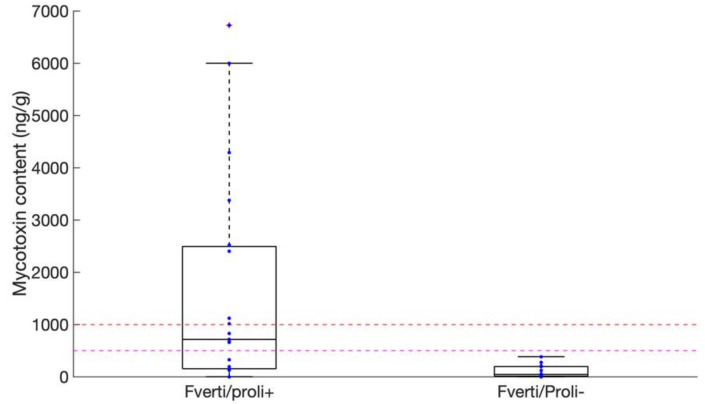
Boxplot representation of *Fumonisin* contents of *Fum1*-positive samples that tested positive for *F. verticillioides*, and *F. proliferatum* for five of them out of 18 (“Fverti/proli+”), or negative (“Fverti/proli-”). The pink and red dashed lines indicate the 500 and 1000 ng·g^−1^ thresholds, respectively.

Molecular detection of *Fusarium* species (see Materials and Methods) indicates that *Fusarium verticillioides*, a worldwide spread fumonisin-producer previously identified as a major contaminant of Peruvian corn [18], was detected in 18 samples out of the 39 ones positive for *Fum1*, 17 samples of corn and one sample of wheat. In the 21 samples negative for the presence of *F. verticillioides*, one was positive for the fumonisin producer *F. proliferatum*, which was also present as a co-contaminant in five of the 18 samples polluted with *F. verticillioides*. Measured levels of contamination with Fumonisins were compared between samples positive for *F.* spp. *verticillioides* and/or *proliferatum*, or negative (Figure 5). Our observations suggest that the highest levels of contaminations may be ascribed to the presence of *F. verticillioides* and, more anecdotally, of *F. proliferatum*.

### 2.3. Assessing Contaminations with Trichothecenes and Zearalenone as well as Their Corresponding Producing Fusarium species in Crop Samples

**Figure 6:**
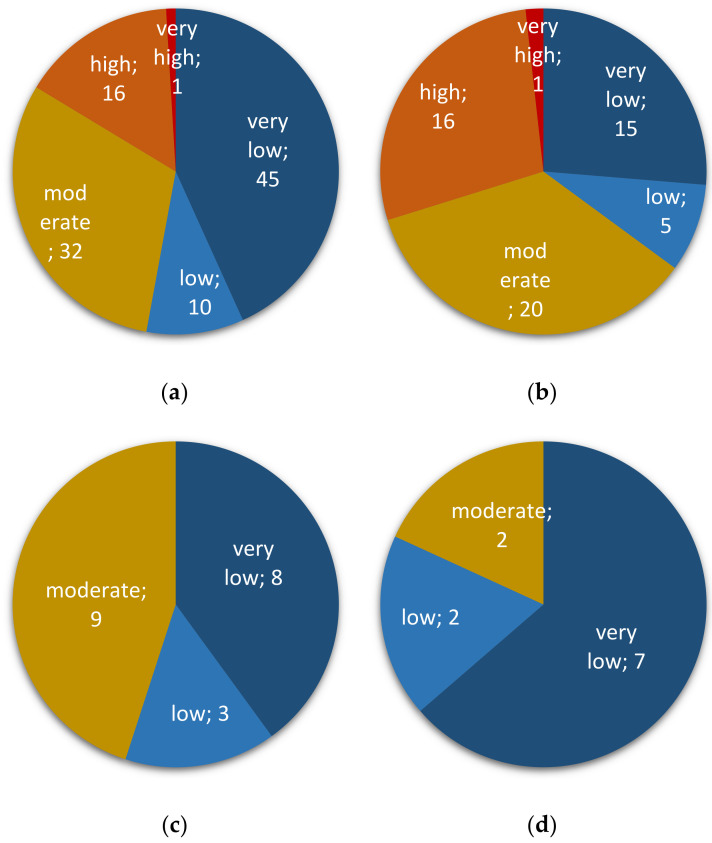
Type B trichothecene (TCTB) risk assessment in collected cereal and pseudo-cereal grains. Risk is ranked as *very low*, *low*, *moderate*, *high*, or *very high*. Number after semicolon indicates corresponding sample size. (**a**) all sample types; (**b**) corn; (**c**) wheat; (**d**) amaranth seeds.

Similar to the analysis performed for Fumonisin-associated risk evaluation, we assessed the risk of having mycotoxins belonging to the family of trichothecenes in food samples (*low*, *moderate*, *high,* or *very high*) by the quantitative detection of the gene *Tri5*, mandatory for their biosynthesis [19]. Two alleles of *Tri5* were searched for (see Material and Methods, Supplementary Materials and Methods, and Table S1): one found in producers of type A trichothecenes (*Tri5*-TCTA) and one found in producers of type B trichothecenes (*Tri5*-TCTB). All samples were negative for *Tri5*-TCTA, indicating the likely absence of these toxins as contaminants of Peruvian samples (data not shown). On the other hand, the allele *Tri5*-TCTB was detected in 59 samples out of the 104 that were positive for the presence of *Fusarium* spp. (57% of them; Figure 6a): nearly 74% of the corn samples (42 out of 57; Figure 6b), 60% of the wheat samples (12 out of 20; Figure 6c), over a third of the amaranth samples positive for *Fusarium* presence (four out of 11; Figure 6d), and one sample of oat (out of four; data not shown). None of the two barley samples or of the 10 rice samples tested positive for *Tri5-TCTB*. All crops considered, TCTB-associated risk seems to come mostly from corn samples, followed by wheat to a lesser extent, two major staples of the Peruvian diet.

Samples with TCTB risk categorized *moderate* to *very high*, i.e., 49 samples (37 samples of corn, nine of wheat, two of amaranth seeds, and one of oat) were further analyzed for the presence of the TCTB members DON and its acetylated form (15- and 3-ADON) as well as nivalenol (NIV) and its acetylated form (FX) by analytical methods (HPLC-MS, see Materials and Methods). A total of 25 samples (19 of corn, and six of wheat) were positive for DON (accompanied or not by 15-acetylated form) and three samples of corn contained NIV (with or without FX), all of them well below the 750 ng·g^−1^ European limit (EC 1881/2006) of maximum admissible level for cereal flour and meal destined to human consumption (Figure S4a). A significant positive Pearson correlation (estimated R^2^ = 0.505 and *p-value* = 0.0002) could be detected between TCTB content and the relative abundance of *Tri5*-containing DNA measured by qPCR (Figure S4b). Considering that TCTB producers can also produce the mycotoxin zearalenone, this toxin was further searched for in the 49 samples positive for *Tri5*-TCTB with *moderate* to *very high*-risk levels (HPLC-MS, see Materials and Methods). A total of 11 samples contained zearalenone (one of amaranth seeds sample and 10 of corn), all but one corn sample being below the 100 ng·g^−1^ European limit of maximum admissible level for unprocessed cereals or corn destined to direct human consumption (EC 1881/2006), and another one nearing that threshold (Figure S4c). As a whole, these observations indicate that *Fusarium* species that can produce TCTB and zearalenone could develop on our Peruvian cereal samples but productions of DON, NIV, and/or acetylated forms as well as ZEN were not favored.

Among the *Fusarium* species that can produce TCTB, *F. graminearum* is one of the most often encountered worldwide as well as, to a lesser extent, *F. pseudograminearum*, *F. culmorum*, and *F. cerealis* [8,20]. Using species-specific primers (see Materials and Methods), the presence of *F. graminearum* was detected in 78% of the samples (*n* = 46), while *F. pseudograminearum* could not be detected in any of the 59 samples that previously tested positive for *Tri5*-TCTB (data not shown). Co-occurring species were detected in three of these samples: *F. culmorum* in one corn sample containing NIV/FX (62 ng·g^−1^), and *F. cerealis* in the two remaining (corn and wheat) samples containing DON/15-ADON. *F. cerealis* was further detected alone in one corn sample, and *F. culmorum* alone in the oat sample (both with no detectable toxins). Measured levels of contamination with TCTB were compared between samples spoiled or not spoiled with *F. graminearum* (Figure S5). Although of low intensity, TCTB-content distribution seems to trend towards the higher range of values when *F. graminearum* is detected.

### 2.4. Frequencies of Co-Contaminations with Fusarium Species and The Mycotoxins Fumonisins, Trichothecenes, and Zearalenone

**Figure 7:**
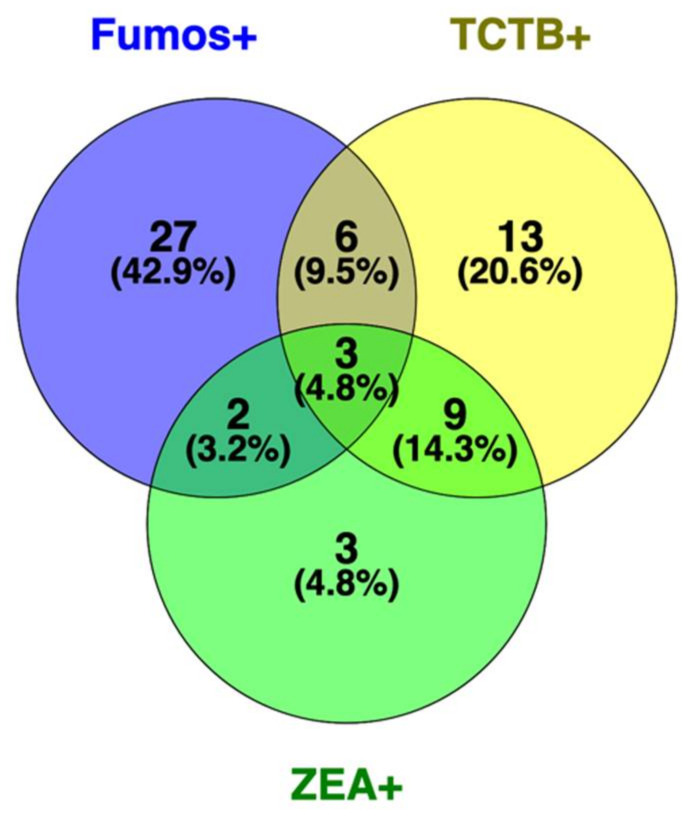
Multiple contaminations in collected cereal and pseudo-cereal grains. Venn diagram (prepared with Venny 2.1.0 [21]) of sample counts considering contaminations with Fumonisins (Fumos+), TCTB (TCTB+), and zearalenone (ZEA+). All sample types are considered.

Beyond absolute levels of contamination of crops within a given family of mycotoxins, co-occurrences of different types of toxic metabolites raise another flag for food safety due to possible cumulative and synergistic toxic effects, even at levels below maximum tolerable individual threshold especially regarding chronic dietary exposure (a summary of mycotoxin content in corn and wheat is proposed; Figure S6). We combined all toxin analysis results to evaluate how often such multiple contaminations may occur. Results are displayed in Figure 7. Nearly 32% of the samples (20 out of the 63 positives for at least one of the toxins Fumonisins, TCTB, or zearalenone) are contaminated with at least two different families of mycotoxins.

## 3. Discussion

Mycotoxins are a major threat to food safety worldwide. Cereals are known to be particularly prone to contaminations, including corn. Our analysis shows that nearly one sample of Peruvian corn out of six was contaminated with very high levels of Fumonisins, well exceeding the 1000 ng·g^−1^ maximum admissible level for safe human consumption, as set by the EU. Provisional maximum tolerable daily intake (PMTDI) for Fumonisins was evaluated by the Joint FAO/WHO Expert Committee on Food Additives (JECFA) in 2001, 2011, and 2016 [22,23,24] based on toxicological assessments. PMTDI was set at 2 µg/kg of body weight (bw) per day for Fumonisins B1, B2, and B3, alone or in combination. Actual national intake values largely depend on the diet and may well exceed this PMTDI in some regions of the world, especially if their diet contains high amounts of corn [25]. In Guatemala, for example, exposure to Fumonisins can be regarded as worrisome, with an estimated daily intake of up to 23 µg/kg/bw/day [26,27], i.e., well above the defined 2 µg/kg/bw/day. In Peru, a high risk of excessive exposure to Fumonisins was previously highlighted in purple corn, used for the preparation of a widely consumed beverage called *chicha morada* [28]. This particular variety showed very high levels of contaminations with Fumonisins, i.e., 2,586 ng·g^−1^ in average and peaking at 27,490 ng·g^−1^, with frequent cases of co-contaminations with aflatoxins mostly below the 10 ng·g^−1^ maximum admissible level for safe human consumption set by E.U. (or 5 ng·g^−1^ for AFB1 only).

In addition to these canonical mycotoxins, the presence of “masked mycotoxins”, i.e., mycotoxins biologically modified by the plant thus having modified toxicities and analytical detectability [29], should be taken into account in exposure assessments. This consideration is particularly relevant for Fumonisin B1, for which up to 40% of the total FB1 content of a maize sample was found associated with the matrix [30] with the possibility of subsequent release of FB1 during processing and/or after ingestion [3]. Considering corn is highly consumed in various forms in the Peruvian-type diet and the frequent contaminations of corn kernels described in the present study, exposure of the Peruvian population to Fumonisins may well exceed the PMTDI, which raises a serious health concern. This concern may be exemplified by our frequent observation of contaminations with more than one family of mycotoxins, i.e., any combination of Fumonisins, TCTB, and zearalenone, and the possible consequences due to possible cumulative and/or synergistic toxic effects.

This question of food contamination of Peruvian (pseudo)-cereals with *Fusarium* mycotoxins has some parallels in current health concerns observed in the local population. For example, the occurrence of early-age forms of liver cancer has been persistently described in patients from Peru, and more broadly, in South America [31,32]. This clinical presentation is associated with a peculiar mutation spectrum and the presence of hepatic foci of cellular alteration within the liver parenchyma that could be a hallmark of chronic liver insults caused by exposure to genotoxic substances, as observed in rodent animal models [33,34,35,36]. The potential brunt of dietary mycotoxin exposure has thus been suggested to intervene as a cofactor in this phenomenon [37,38]. Such harmful effects induced by Fumonisin B1 produced by *Fusarium* have been previously monitored on rat liver [39].

We found that, in the samples tested here, the presence of high levels of Fumonisins could be mostly ascribed to the presence of *F. verticillioides* (~46% of the samples with detectable levels of Fumonisins). The fungi responsible for the presence of Fumonisins in the 54% of less contaminated samples could, however, not be determined as belonging to either of the species *proliferatum* or *verticillioides*. A single study identified *Fusarium subglutinans*, a phylum later split into *F. subglutinans* and the closely related species *Fusarium temperatum* [40,41], and *Fusarium moniliforme* (later on renamed as *F. verticillioides*) as two major contaminants of Peruvian maize kernels [18]. Both *F. subglutinans* and *F. temperatum* have been previously reported to produce Fumonisins in corn grown in various countries [41,42]. In their previous assessment in Peru, Logrieco and colleagues found the strains of *F. subglutinans* they isolated to primarily produce beauvericin and moniliformin, two mycotoxins qualified as “emerging”, i.e., mycotoxins that are “neither routinely determined, nor legislatively regulated” with, however, “the evidence of their incidence is rapidly increasing” [43]. In line with these results, in Argentina, *F. temperatum* was also detected as a frequent contaminant of maize kernels but isolated strains largely produced beauvericin and only a few of them produced Fumonisins [44,45]. Consistent monitoring across several years would be required considering that Peru is subjected to extreme climatic events, notably El Niño/La Niña events, that are prime factors for contaminations with mycotoxins [46]. A 10-year survey was previously conducted on corn from South America from 2008 to 2017 and documented a somehow increasing trend for the years 2012 onward [13]. Further fungal contaminant profiling of Peruvian corn kernels could provide elements to refine the potential risk associated with mycotoxins.

A control strategy to reduce contamination of foodstuffs with Fumonisins requires a multifaceted approach combining pre- and post-harvest interventions. Increased knowledge on environmental factors that promote *F. verticillioides* infection and the production of Fumonisins in grains led to the definition of Good Agricultural Practices that include selection of tolerant genotypes, early timing for planting, adequate irrigation to avoid drought stress, control of insects, and biocontrol strategies [47]. In low- and middle-income countries where technology and infrastructures, e.g., grain storage facilities, may not always be adapted to the previous methods, ensuring low exposure to Fumonisins remains a significant challenge. In addition to investments in infrastructures, pre- and postharvest technologies, and robust monitoring that would allow early warning and implementation of remediation plans, great emphasis should be put on promoting increased public knowledge and awareness.

## 4. Materials and Methods

### 4.1. Access to Genetic Resources and Benefit-Sharing

The present study was carried out in strict accordance with the principles contained in the Nagoya Protocol on Access to Genetic Resources and the Fair and Equitable Sharing of Benefits Arising from their Utilization to the Convention on Biological Diversity. A competent legal authority, i.e., the Peruvian National Institute for Agrarian Innovation (INIA), issued biodiversity collection permits for access to the cultivated genetic resources and their use for the present research (Biodiversity Collection Permits Numbers 375-2016-INIA-J/DGIA, 621-2016-INIA-J/DGIA, and 021-2018-MINAGRI-INIA-DGIA/SDRIA).

### 4.2. Experimental Design and Sampling

A total of 119 samples (about 200 g each) were collected through donations and/or purchases in various local farmers’ markets in Peru (Table 1). Samples were selected to reflect the cereal dietary of the Peruvian population, as documented in a survey based on a semi-structured questionnaire [48]. Upon reception, each sample was ground into a fine powder (final fineness < 40 µm) using an ultra-centrifugal mill ZM200 (RETSCH) and stored at 4 °C in air-tight containers for up to six months until further processing.

### 4.3. DNA Extraction

DNA was purified from 50 mg of ground sample using NucleoMag^®^ Plant kit (Macherey-Nagel, Hoerdt, France). Briefly, ground samples were homogenized with a Precellys^®^ Evolution (Bertin Instruments, Montigny-le-Bretonneux, France) for 2 × 30s (with a 5 s break) at 6500 rpm. Homogenized samples were mixed with 500 µL lysis Buffer MC 1 and 10 µL RNase A, and incubated at 56 °C for 30 min. After clearing the lysate by centrifugation (16,000 g for 20 min at room temperature), 100 µL of the clear lysate was transferred into a well of a separation plate containing 15 µL of magnetic beads diluted in 100 µL of buffer MC 2. All subsequent purification steps taken were automated in this plate in a MagMAX^TM^ express (Applied Biosystems by Thermo Fisher Scientific, Paisley, U.K.) system. After mixing and incubating 5 min at room temperature, the magnetic beads with DNA bound on their surface were collected and washed for 5 min at room temperature in 200 µL of MC 3 buffer, followed by a second wash in 200 µL of MC 4 buffer (5 min at room temperature with constant mixing), and the last wash in 200 µL of freshly made 80% ethanol (5 min at room temperature with constant mixing). While beads on the magnet, a final 1 min-long wash was performed in MC 5 buffer before eluting DNA in 80 µL of MC 6 buffer (5 min at room temperature with constant mixing). DNA quality and quantity were assessed by 1% agarose gel electrophoresis and UV spectrophotometry, respectively.

### 4.4. Fusarium Contaminations and Mycotoxin Risk Assessment by qPCR Assays

Analyses were performed using 20 ng of each DNA sample mixed in a 10 μL-reaction volume, using the QuantiFast^TM^ SYBR^®^ Green PCR kit (Qiagen, Courtaboeuf, France) and primers in Table 2. Reactions were carried out on a LightCycler^®^ LC2.0 system equipped with the LightCycler software 3.5.3 (Roche Diagnostics France, Meylan, France) set up with the cycling and melting curve analysis conditions [five minutes at 95 °C; 40×(10 s at 95 °C; 40s at annealing temperature); 0 s at 95 °C; 15 s at 65 °C; 0 s at 95 °C; 30 s at 40 °C]. Fungal DNA abundance was estimated by external calibration using gDNA extracted from axenic cultures of *Fusarium* species (see Table 2), which were also used as a reference for melting curve inspection to ensure amplification specificity. qPCR results obtained for target genes were used to qualify the corresponding mycotoxin-associated risks as *very low* (no amplification, i.e., not detected), *low* (35 < Cq ≤ 40, i.e., detected but not quantifiable), *moderate* (30 < Cq ≤ 35), *high* (25 < Cq ≤ 30), or *very high* (Cq ≤ 25).

The presence of *Fusarium* spp. was assayed by testing for the presence of the gene encoding tubulin beta chain using broad-range primers ßT2a [49] and ßt-2 [50] (Table 2). The risk level of having samples contaminated with *Fusarium* mycotoxins was estimated with the detection of genes involved in their biosynthetic pathways: *Fum1* for Fumonisins, Type B and type A *Tri5* alleles for type B and type A trichothecenes, respectively (see Supplementary Methods and Table S1). The identification of contaminant *Fusarium* species that can produce the mycotoxins for which samples were found at risk was sought using species-specific primers (Table 2). Species-specific assays were further used to putatively attribute contaminations to the presence of given *Fusarium* species (see Table 2).

### 4.5. Mycotoxin Content Analysis

Ground samples were used as starting materials. The analysis of fumonisin content (FB1, FB2, and FB3) was performed according to the method published by Picot and Colleagues [56]. TCTB and zearalenone contents were analyzed using LC-MS/MS with procedures adapted from previously published methods [57,58]. Briefly, mycotoxins were extracted from one gram of finely ground samples with 5 mL of an acetonitrile/water mixture (84/16, *v*/*v*). After 1 h of agitation, 4 mL were evaporated to dryness at 50 °C under a gentle stream of nitrogen. Samples were reconstituted in 200 µL of methanol/water (50/50, *v*/*v*) before analysis. MS analysis was performed using a QTrap 2000 system (Sciex, Villebond sur Yvette, France) equipped with an ElectroSpray Ionization (ESI) source and an 1100 Series HPLC system (Agilent, Les Ulis, France). Chromatographic separation was achieved on a Kinetex XB - C18 100 Å column (150 × 4.60 mm, 2.6 μm) (Phenomenex, Torrance, CA, USA) protected with a guard column of the same material and maintained at 45 °C. Solvent A consisted of methanol/water (10/90, *v*/*v*) and solvent B consisted of methanol/water (90/10, *v*/*v*). Gradient elution was performed with the following conditions: 4 min with a linear gradient from 85% to 5% A, 4 min held at 5% A, 1 min linear gradient from 5% to 85% A and 85% A for 8 min post run reconditioning. The injection volume was 5 μL. The flow rate was kept at 0.7 mL/min and a split was used so that 350 µL/min was forwarded to the ESI source. The electrospray interface was used in the negative ion mode at 400 °C with the following settings: curtain gas, 20 p.s.i.; nebulizer gas, 30 p.s.i.; auxiliary gas, 70 p.s.i.; ion spray voltage, −4200 V; declustering potential, −30 V; entrance potential, −10 V. Quantification was performed using external calibration ranging from 10 to 1000 ng/mL prepared with pure standards (Romers Lab, Tulln, Austria).

## Figures and Tables

**Table 1:** Summary of metadata for the collected samples.

Type of Grain	Number of Samples	Landscape of Origin		
		*Costa*	*Sierra*	*Selva*
corn	60	7	51	2
wheat	25	1	24	0
amaranth	12	0	12	0
rice	12	4	5	3
barley	6	0	6	0
oat	4	2	2	0
Total	119	14	100	5

**Table 2:** Primers used in this study.

Primer Names	Primer Sequences	Ta ^1^ (°C)	*Fusarium* Species/Toxin Producers Targeted	References
ßtub2a ßt-2	GGTAACCAAATCGGTGCTGCTTTC GATTGACCGAAAACGAAGTTG	58	Broad range ^2^	[49,50,51]
Fum1-F Fum1-R	GGATTGGCTGGATCTTCACAG GAAGATGGCATTGATTGCCTC	57	Fumonisins ^3^	[52]
Tri5-TCTA-F Tri5-TCTA-R	CTATTCCTTGAGATTACAT CCTTGTAGAATGACATAAGA	58	TCTA ^4^	This paper
Tri5-TCTB-F Tri5-TCTB-R	GATGGACACGATTGAGTG GCTCAAAGAACTTGCAGA	58	TCTB ^5^	This paper
Fum1-656F Fum1-1158R	CGGTTGTTCATCATCTCTGA GCTCCCGATGTAGAGCTTCTT	60	*F. verticillioides*	[53]
Fum1-656F Fum1-872R	TGCTCGTCATCCCTGATAG GAAGATGGCATTGATTGCCTC	60	*F. proliferatum*	[52]
Fg16N-F Fg16N-R	ACAGATGACAAGATTCAGGCACA TTCTTTGACATCTGTTCAACCCA	60	*F. graminearum*	[54]
FC01-F FC01-R	ATGGTGAACTCGTCGTGGC CCCTTCTTACGCCAATCTCG	60	*F. culmorum*	[54]
FcroA-F FcroA-R	CTCAGTGTCCACCGCGTTGCGTAG CTCAGTGTCCCAATCAAATAGTCC	62	*F. cerealis*	[54]
Fp1-F Fp1-R	CGGGGTAGTTTCACATTTC(C/T)G GAGAATGTGATGA(C/G)GACAATA	57	*F. pseudograminearum*	[55]

^1^ Annealing temperature; ^2^ Verified for the species *acuminatum*, *asiaticum*, *austroamericanum*, *avenaceum*, *boothii*, *cerealis*, *compactum*, *cortaderiae*, *culmorum*, *equiseti*, *fujikuroi*, *graminearum*, *langsethiae*, *lateritium*, *lunulosporum*, *meridionale*, *mesoamericanum*, *oxysporum*, *poae*, *pseudograminerum*, *sacchari*, *sambucinum*, *sporotrichioides*, *subglutinans*, *tricinctum*, *temperatum*, *verticillioides*, *venenatum*; ^3^ Verified for the species *F. verticillioides*, *F. proliferatum*, and *F. fujikuroi*; ^4^ Verified for the species *acuminatum*, *equiseti*, *langsethiae*, *poae*, *sambucinum*, *sporotrichioides*; ^5^ Verified for the species *asiaticum*, *austroamericanum*, *boothii*, *cerealis*, *cortaderiae*, *culmorum*, *graminearum*, *lateritium*, *lunulosporum*, *meridionale*, *mesoamericanum*, *pseudograminerum*.

## Data Availability

The data presented in this study are available on request from the corresponding author.

## Supplementary Materials

The following are available online at https://www.mdpi.com/2072-6651/13/2/172/s1, Figure S1: *Fusarium* risk assessment in collected amaranth, barley, and oat grain samples, Figure S2: Assessment of the risk associated with the presence of fumonisin producers in cereal and pseudo-cereal grains contaminated with *Fusarium* species, Figure S3: Fumonisin content as a function of the relative abundance of *Fum1*-containing DNA measured by qPCR in cereal samples, Figure S4: Toxin contamination profiles in cereal and pseudo-cereal grains contaminated with *Fusarium* species that can produce TCTB and zearalenone, Figure S5: Boxplot representation of TCTB contents of *Tri5*-positive samples that tested positive for *F. graminearum* (“Fg+”) or negative (“Fg-”), Figure S6: Boxplot representation of mycotoxin content in corn and wheat measured in samples with moderate to very high mycotoxin-associated risk, Table S1: *Fusarium* species and NCBI sequence accession numbers used to design TCTA and TCTB-discerning primers.
