# Supplementary material for: Natural Occurrence of Mycotoxin-Producing Fusaria in Market-Bought Peruvian Cereals: A Food Safety Threat for Andean Populations
Source: Toxins (Basel). 2021 Feb 23;13(2):172. doi: 10.3390/toxins13020172 (PMC7926892; doi:10.3390/toxins13020172)
Supplement: Supplementary file 1 [file toxins-13-00172-s001.pdf]

toxins

MDPI

Natural Occurrence of Mycotoxin-Producing *Fusaria* in Market-Bought Peruvian Crops: a Food Safety Threat for Andean Populations

Christine Ducos, Laetitia Pinson-Gadais, Sylvain Chereau, Florence Richard-Forget, Pedro Vásquez-Ocmín, Juan Pablo Cerapio, Sandro Casavilca-Zambrano, Eloy Ruiz, Pascal Pineau, Stéphane Bertani and Nadia Ponts

1. Supplementary Methods: Tri5-TCTA and Tri5-TCTB primer design

Multiple sequence alignment was performed using nucleotide sequences of *Tri5* gene extracted from *Fusarium* species that can produce either type A trichothecens (TCTA) or type B trichothecens (TCTB). Species and accession numbers are provided in Supplementary Table S1. Primers for Sybr Green-based qPCR assay were subsequently designed in discerning regions using Primer3 [1,2].

Table S1. *Fusarium* species and NCBI sequence accession numbers used to design TCTA and TCTB-discerning primers.

Toxins Produced	Species and Strain	NCBI Accession Number of *Tri5* Sequence
TCTA	*F. camptoceras* NRRL3381	GQ915545.1
	F. sporotrichioides	AY130293.1
	F. langsethiae IBT 9956	AF449792.1
	F. kyushuense NRRL 25349	GQ915548.1
	F. incarnatum NRRL 31160	GQ915550.1
	F. armeniacum FRC R-0933	GQ915543.1
	G. publicaris FRC_R 07843	GQ915552.1
TCTB	*F. graminearum* NRRL9394	AY102605.1
	F. austroamericanum NRRL 2903	AY102596.1
	F. asiaticum NRRL 28720	AY102590.1
	F. graminearum GZ3639	AF359361.3
	F. graminearum PH-1	supercont3.2 -3376420-3377787 [1]
	F. cortaderiae NRRL 29306	AY102601.1
	F. culmorum NRRL 25475	AY102571.1
	F. acaciae-mearnsii NRRL 26754	AY102577.1
	F. cerealis NRRL 25491	AY102572.1

[1] Sequence source from [3].

2. Supplementary Figures

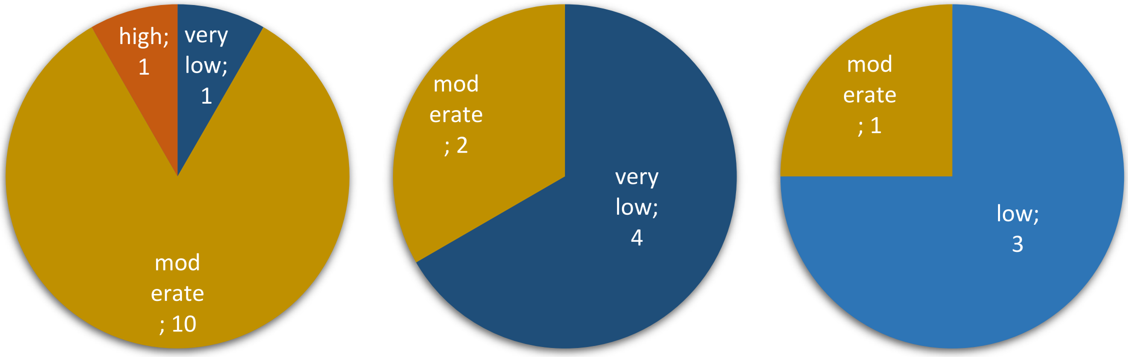

(**a**) (**b**) (**c**)

Figure S1. *Fusarium* risk assessment in collected amaranth, barley and oat grain samples. Risk is ranked as *very low*, *low*, *moderate*, *high*, or *very high*. Number after semicolon indicates corresponding sample size. (**a**) amaranth seeds; (**b**) barley; (**c**) oat.

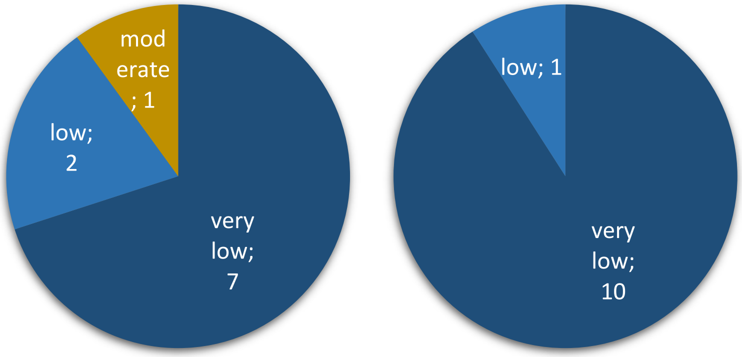

(**a**) (**b**)

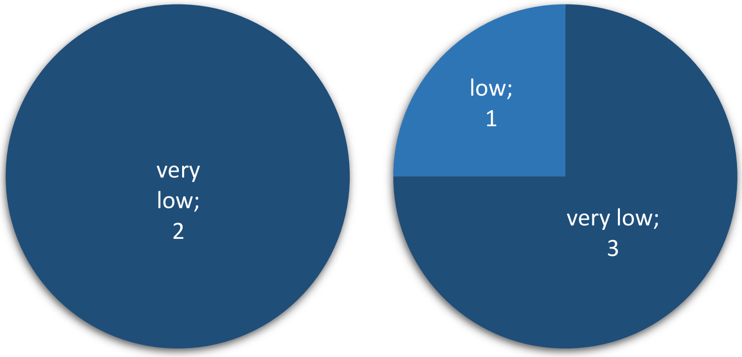

(**c**) (**d**)

Figure S2. Assessment of the risk associated with the presence of fumonisin producers in cereal and pseudo-cereal grains contaminated with *Fusarium* species. Risk is ranked as *very low*, *low*, *moderate*, *high*, or *very high*. Number after semicolon indicates corresponding sample size. (**a**) rice; (**b**) amaranth seeds; (**c**) barley; (**d**) oat.

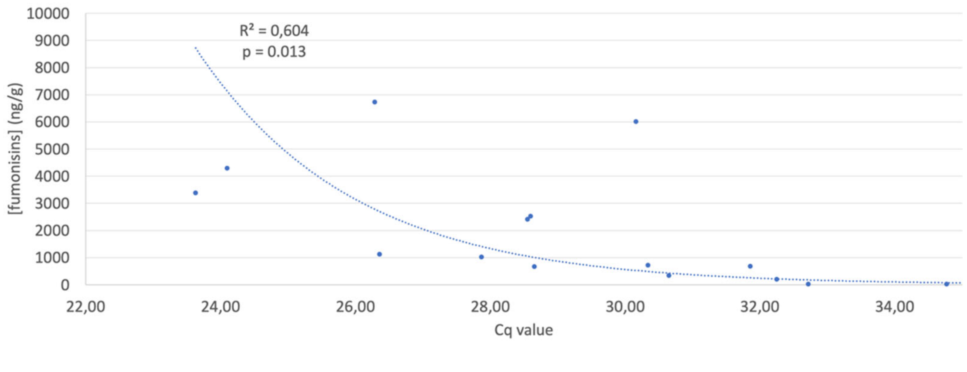

Figure S3. Fumonisin content as a function of the relative abundance of *Fum1*-containing DNA measured by qPCR in cereal samples. Pearson product-moment correlation coefficient (R^2) and associated *p*-value were computed with Matlab 2020b (MathWorks) and used to estimate the goodness of fit of the monotonic non-linear relationship.

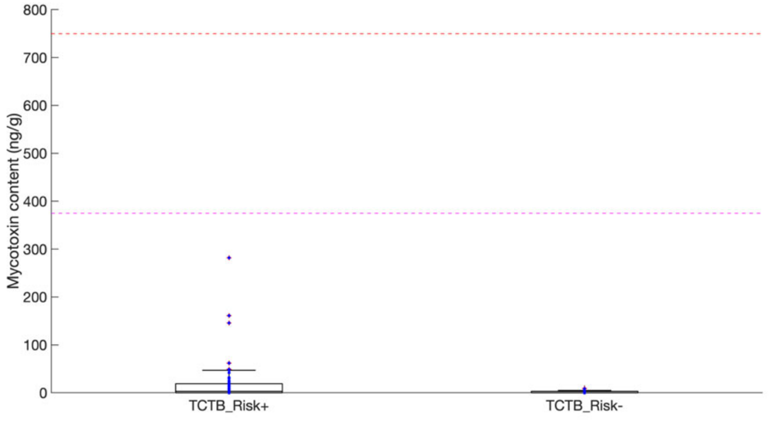

Mycotoxin content (ng/g)
800
700
600
500
400
300
200
100
0
TCTB_Risk+
TCTB_Risk-

(**a**)

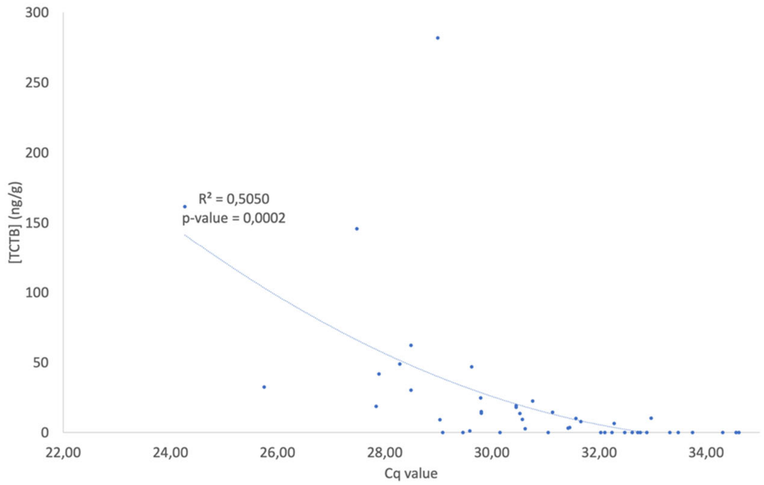

[TCTB] (ng/g)
300
250
200
150
100
50
0
R² = 0,5050
p-value = 0,0002
22,00
24,00
26,00
28,00
30,00
32,00
34,00
Cq value

(**b**)

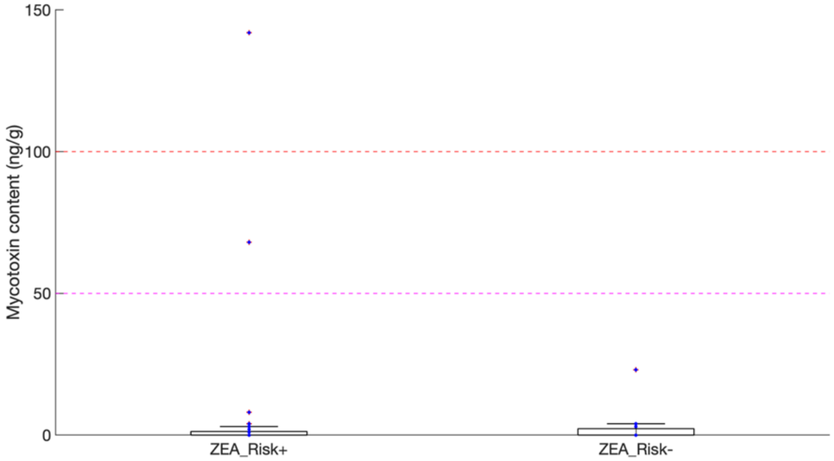

Mycotoxin content (ng/g)
150
100
50
0
ZEA_Risk+
ZEA_Risk-

(**c**)

Figure S4. Toxin contamination profiles in cereal and pseudo-cereal grains contaminated with *Fusarium* species that can produce TCTB and zearalenone. (**a**) Boxplot representation of TCTB contents. "TCTB-Risk+" refers to samples with *moderate* to *very high* TCTB-associated risk, and "TCTB-Risk-" refers to 10 randomly selected samples with risk ranked as *very low* to *low*. The pink and red dashed lines indicates the 375 and 750 ng.g-1 thresholds, respectively; (**b**) TCTB content as a function of the relative abundance of *Tri5*-containing DNA measured by qPCR in cereal samples. Pearson product-moment correlation coefficient (R^2) and associated *p*-value were computed with Matlab 2020b (MathWorks) and used to estimate the goodness of fit of the monotonic non-linear relationship; (**c**) Boxplot representation of zearalenone contents. "ZEA-Risk+" refers to samples with *moderate* to *very high* ZEA-associated risk, and "ZEA-Risk-" refers to samples with risk ranked as *very low* to *low*. The pink and red dashed lines indicates the 50 and 100 ng.g^{-1} thresholds, respectively.

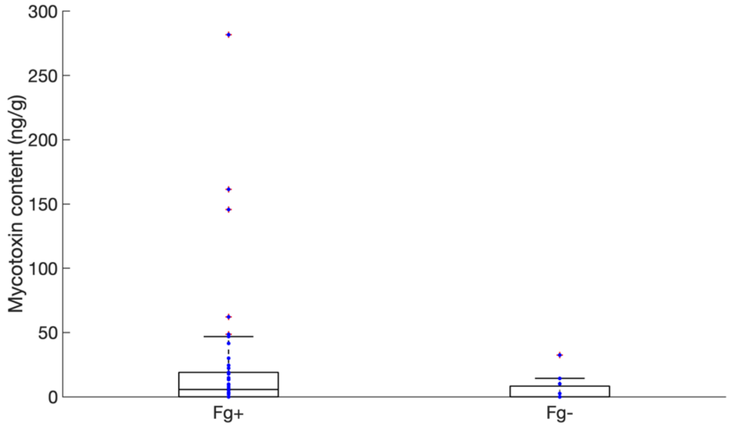

Figure S5. Boxplot representation of TCTB contents of *Tri5*-positive samples that tested positive for *F. graminearum* ("Fg+") or negative ("Fg-").

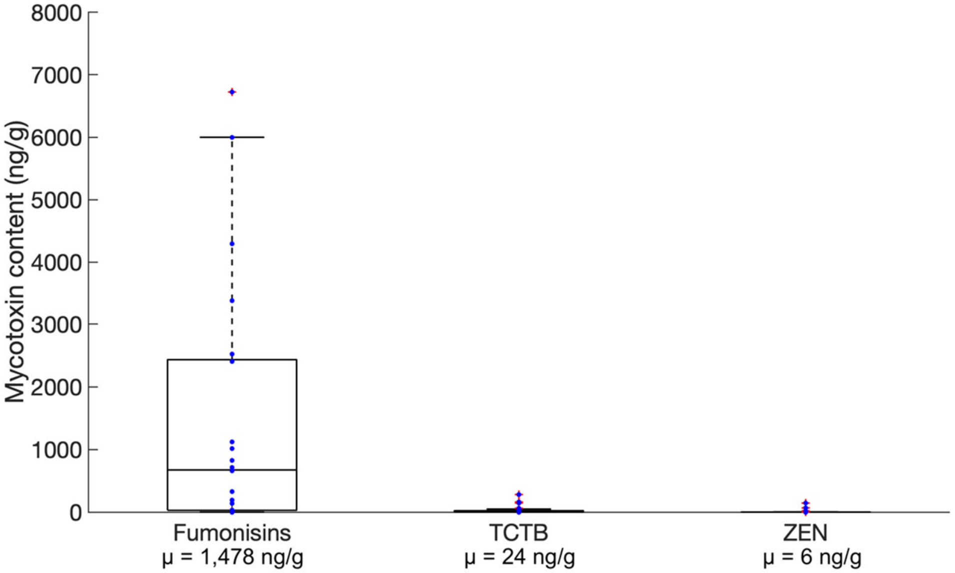

(**a**)

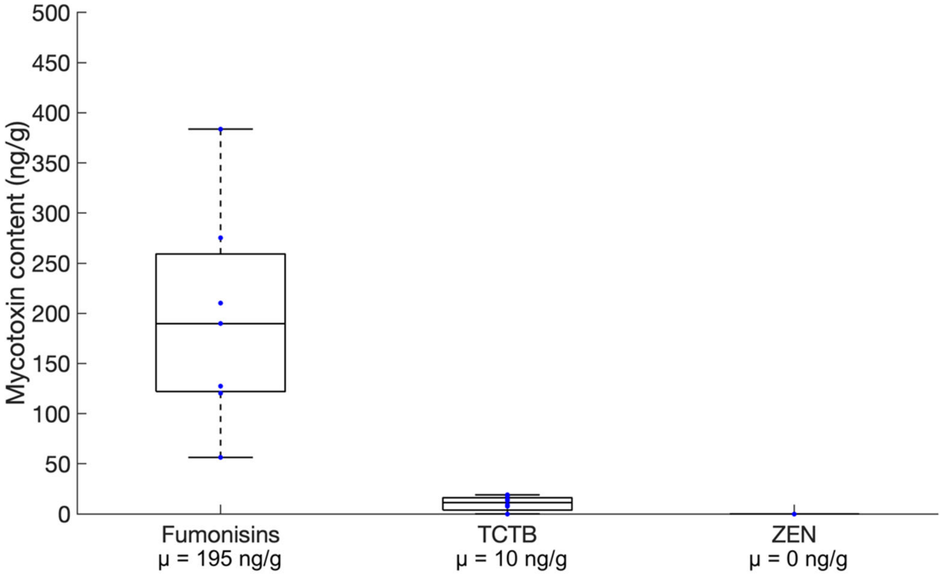

(**b**)

Figure S6. Boxplot representation of mycotoxin content in corn and wheat measured in samples with *moderate* to *very high* mycotoxin-associated risk. The values μ indicate mean values for the corresponding toxins. μ = 0 means no mycotoxin could be detected. (**a**) Corn. (**b**) Wheat. Boxplots and means were not computed for other cereals for which only three samples or less were at *moderate* to *very high* mycotoxin-associated risk and showing very low or no detectable levels of mycotoxins.

3. Additional references

1. Untergasser, A.; Cutcutache, I.; Koressaar, T.; Ye, J.; Faircloth, B.C.; Remm, M.; Rozen, S.G. Primer3—New Capabilities and Interfaces. Nucleic Acids Res **2012**, 40, e115, doi:10.1093/nar/gks596.

2. Koressaar, T.; Remm, M. Enhancements and Modifications of Primer Design Program Primer3. Bioinformatics **2007**, 23, 1289–1291, doi:10.1093/bioinformatics/btm091.

3. Wong, P.; Walter, M.; Lee, W.; Mannhaupt, G.; Münsterkötter, M.; Mewes, H.-W.; Adam, G.; Güldener, U. FGDB: Revisiting the Genome Annotation of the Plant Pathogen Fusarium Graminearum. Nucleic Acids Res **2011**, 39, D637-639, doi:10.1093/nar/gkq1016.
